# Identifying the regional drivers of influenza-like illness in Nova Scotia, Canada, with dominance analysis

**DOI:** 10.1038/s41598-023-37184-z

**Published:** 2023-06-21

**Authors:** Yigit Aydede, Jan Ditzen

**Affiliations:** 1 Saint Mary’s University, Halifax, Canada; 2 Free University of Bolzano, Bolzano, Italy

**Keywords:** Machine learning, Statistical methods, Data mining, Computational models

## Abstract

The spread of viral pathogens is inherently a spatial process. While the temporal aspects of viral spread at the epidemiological level have been increasingly well characterized, the spatial aspects of viral spread are still understudied due to a striking absence of theoretical expectations of how spatial dynamics may impact the temporal dynamics of viral populations. Characterizing the spatial transmission and understanding the factors driving it are important for anticipating local timing of disease incidence and for guiding more informed control strategies. Using a unique data set from Nova Scotia, Canada, the objective of this study is to apply a new novel method that recovers a spatial network of the influenza-like viral spread where the regions in their dominance are identified and ranked. We, then, focus on identifying regional predictors of those dominant regions. Our analysis uncovers 18 key regional drivers among 112 regions, each distinguished by unique community-level vulnerability factors such as demographic and economic characteristics. These findings offer valuable insights for implementing targeted public health interventions and allocating resources effectively.

## Introduction

The recent advances in surveillance systems for infectious disease, capability of data collections and storage, and increased computational resources in the last decade have provided unprecedented tools for the scientific community to understand and, more importantly, combat the spread of infectious disease in populations. The importance of understanding the dynamics of underlying process in viral spread in response to its epidemiological factors such as weather-dependent correlates and most importantly non-pharmaceutical interventions has become more evident with the recent COVID-19 pandemic. While a large number of studies have examined individual-level risk factors for COVID-19, few studies have examined geographic hotspots and community drivers associated with spatial patterns in local transmission.

This study aims to offer insights into the socio-spatial factors impacting the spatial distribution of influenza-like illness by utilizing a novel methodological approach and unique granular data at the FSA level. Through the identification of dominant regional drivers and their associated socio-spatial predictors, our work seeks to contribute to a better understanding of the factors shaping the spatial transmission network. We hope our findings can help inform public health policy discussions, potentially enabling more targeted and effective interventions in addressing infectious diseases like COVID-19 and promoting healthier communities.

Studies investigating outbreaks by social geography provide us invaluable tools for understanding spatial and temporal determinants of the spread. Prior to the COVID-19 outbreak, it has been well-demonstrated that social, geographic, and economic factors impact the rate of infectious disease transmission^1–6^. Socio-spatial influences that have historically contributed to the rapid spread of infections are poor hygiene^6–10^, low income^6,8,11–14^, high population density^6,15^, public and mass transit^7,16,17^, malnutrition^18–20^, and disadvantaged socioeconomic status^21^. There are several recent studies^22–27^ focusing on spatial risk factors associated with the COVID-19 spread. The evidence unambiguously shows that climatic variables, mobility restrictions, and place-based factors like median household income, income inequality, and ethnic diversity in the local population explain a significant spatial variation in COVID-19 incidence. Overall, these studies report strong evidence of the spatial associations between the COVID-19 spread with selected local socioeconomic factors.

This study extends the previous work that investigates how contextual factors can contribute to the spatial distribution of a viral spread in several new directions. Unlike studies using cross-sectional incidence densities, regional hotspots, or spatial clusters (Spatial Scan Statistics^28^), we use a novel method related to a recent literature on granular time series^29^ to explore the formation of spatial dependence in the network of regions. We identify and rank the dominance of each region in the spatial transmission network using the temporal dynamics in the data. With the application of this new method to epidemiological surveillance, we uncover "dominant regional drivers" and associated socio-spatial predictors rather than their associations with regional differences in incidence densities. The set of regional attributes used to identify socio-spatial factors of a spread in earlier studies are very limited due to its availability at a finer spatial scale (i.e., at the postal code). For example, most studies use county-level geographical classifications in the U.S. with few selected variables that are manually identified with a prior knowledge. We use regional data at the FSA (3-digit postal codes—Forward Sorting Area) level with more than 1400 demographic, economic, and social regional variables. Finally, to isolate important space-specific predictors of being a "dominant regional driver" of a viral pathogen, we exploit a framework that provides unbiased conditional variable importance yielded by random forest for feature selection.

## Data

In this study, our focus is on the province of Nova Scotia, Canada, which is situated on the east coast as one of the country's Atlantic provinces. Covering an area of approximately 55,284 square kilometers (21,345 square miles), Nova Scotia ranks as the second-smallest province in Canada. With a population of around one million, a substantial portion resides in the Halifax Regional Municipality (HRM). As the capital city of the province, Halifax serves as a major economic and cultural hub for the region. The climate in Nova Scotia is primarily influenced by its maritime location, resulting in comparatively mild weather conditions throughout the year.

**Figure 1:**
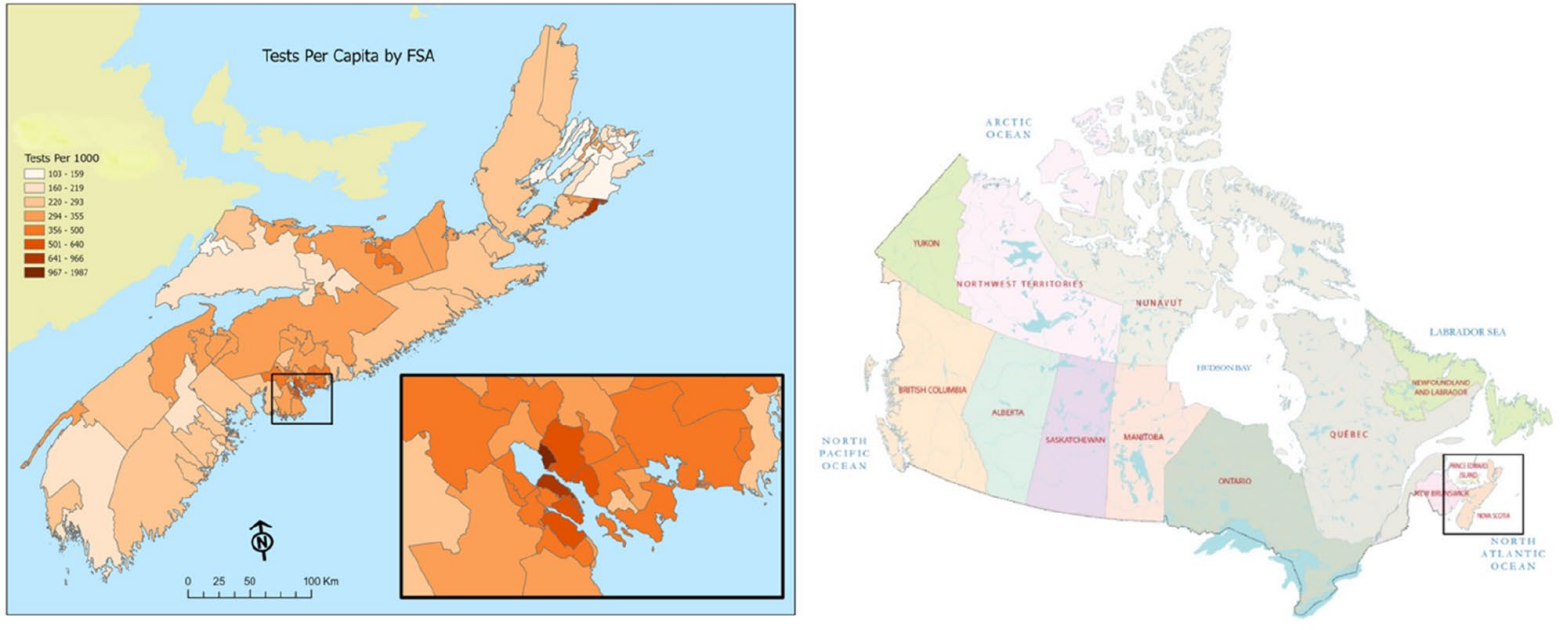
COVID-19 Tests in Nova Scotia, Canada: March-October 2020. *Note*: (1) The magnified section in the first map shows Halifax Regional Municipality (HRM), (2) The first map (left) is created by authors powered by Esri (Canada) and using the map data copyrighted OpenStreetMap contributors and available from https://www.openstreetmap.org. (2) The source of the second map (right) is GISGeography and retrieved from (https://gisgeography.com/canada-map/) (Accessed May 5, 2023).

This study uses a confidential and administrative dataset obtained as a part of Research Nova Scotia (RNS) project (HRC-2020-112) investigating the efficacy of mobility restrictions. The project’s research team selected to the Nova Scotia COVID-19 Research Coalition is the only (as of March 2022) group of researchers that have been permitted to access the data out of Nova Scotia Health Authority (NSHA). We start with a heatmap that summarizes the test numbers for Nova Scotia and Halifax Regional Municipality (HRM) in Fig. 1.

The local public response to the COVID-19 pandemic in Nova Scotia offers a unique dataset akin to controlled clinical trials. Beginning in the first week of March 2020, the local health authority implemented a set of testing guidelines for COVID-19. Individuals experiencing two or more symptoms such as fever (higher than 38C), cough, sore throat, runny nose, and headache were instructed to call 811, which functioned as an initial assessment point operated by registered nurses. The number of daily tests conducted substantially increased, ranging from 700 to 1200, with positivity rates between 1 and 3%. As COVID-19 tests during the first 4 months of the initial wave in 2020 were regulated by the 811 Triage, each referral by the triage represents a significant and accurate level of information about the spatial and temporal distribution of influenza-like illness.

We received the test data in May 2021 containing information by FSA with few unique characteristics: number of tests each day, average age, gender distribution, delay between test and test results, and the test results (positive or negative). The temporal structure of the test data in our study is highly granular, with daily records provided by test centers in each Forward Sortation Area (FSA) in Nova Scotia. Although the COVID-19 case numbers are very rare around 1000 incidents in the first 3 months of the pandemic, this level of detail enables us to comprehensively analyze the spatiotemporal patterns of influenza-like illness within the province.

As a result of the stringent triage process prior to COVID-19 testing, the number of individuals directed to test centers accurately reflects the daily count of symptomatic people who do not have COVID-19 but are experiencing influenza-like illness within each FSA in the province. We employ the incidence density (also known as the incidence rate, calculated as the number of symptomatic individuals/population) from the 1st of March to the 1st of July in 2020 for our analysis. The primary reason for confining our examination to the initial 4 months of the pandemic is the considerable alteration in the testing procedure that took place after this period. As a result, the dataset from the first 4 months serves as a highly focused and precise representation of the spatial and temporal distribution of influenza-like illness.

The data for the socio-spatial risk factors are obtained from Canadian Census Analyser (CHASS) for the year 2016 at the FSA level [Although the 2021 Canadian Census was made available in early 2023, at the time of this study, "Profile of Forward Sortation Areas (FSA)" was not available at Canadian Census Analyser.]. The census profile variables are grouped in 16 subcategories: Population and Dwellings, Age and Sex, Dwelling (dwelling characteristics and household size), Marital Status, Language, Income, Knowledge of Language, Immigration, Aboriginals and Visible Minorities, Housing, Ethnic Origin, Education, Labour, Journey to Work, Language of Work, Mobility. In each category, variables represent averaged values at each FSA and for each gender type. When we include all categories, we obtain more than 1400 socio-spatial variables for each of 112 FSAs in Nova Scotia. Although the richness of data at this level of spatial scale is very desirable, it brings issues due to the curse of dimensionality, which will be addressed later.

## Methods

### Dominant units using graphical models

**Figure 2:**
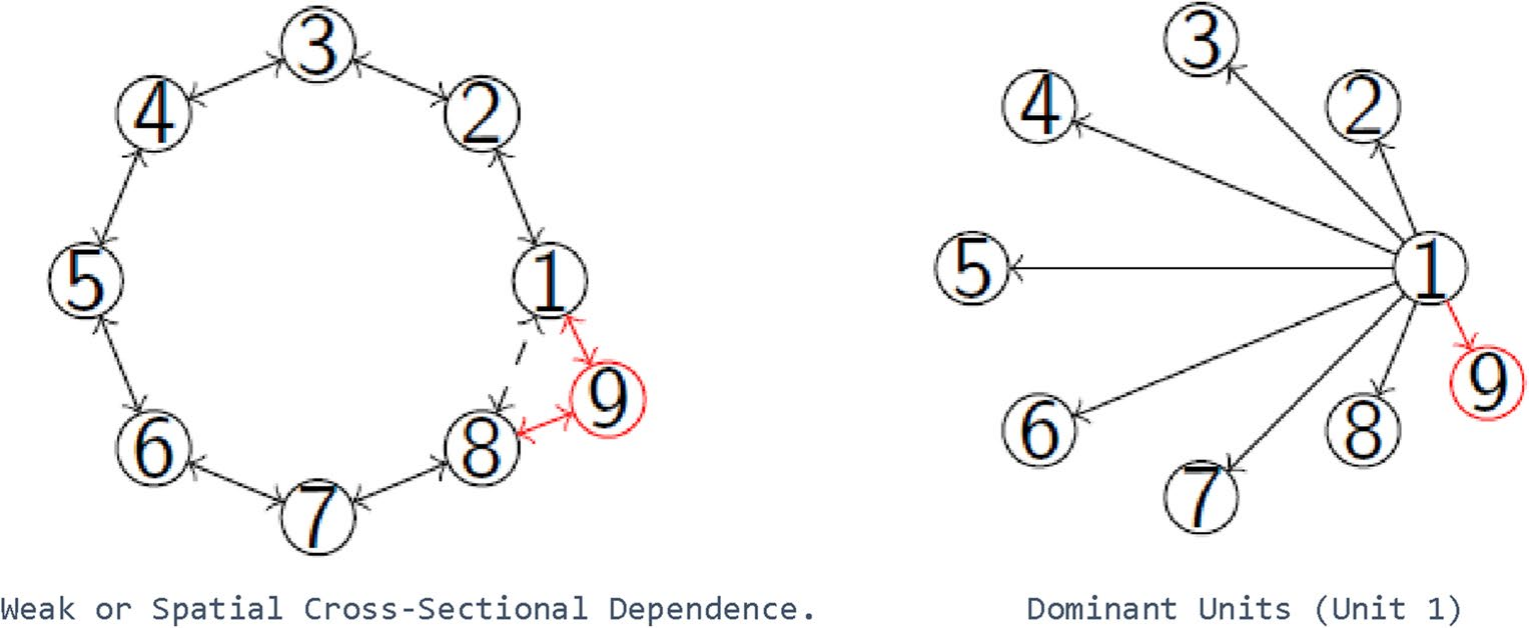
The difference between spatial and network analyses.

Dominant units are units which influence the entire cross-section, that is all other units. In factor models they can often be modelled as observed common factors^29^. The degree a cross-sectional unit influences others varies. If a unit affects only the units closest to it, a shock of such unit will wear out when travelling through the network. This concept is called weak or spatial dependence and usually estimated by spatial methods. An additional unit, marked in red, is connected to two neighbours. The figure on the left in Fig. 2 describes such a setup. Dominant units in turn affect all other units and are at the core of a star type network (on the right): when the dominant unit Number 1 experiences a shock, this shock will influence all other units. Further, if the number of units increases, dominant units will affect those as well.

The identification of dominant units has recently received much attention and can be differentiated into two strands. One based on correlation matrices and the other on measures of connectedness. There are few recent examples for the former^29^ and for the latter Kapetanios et al.^30^ and Pesaran and Yang^31^. Other contributions such as Ditzen and Ravazzolo^32^ or Gumundsson and Brownlees^33^ combine both.

Kapetanios et al.^30^ estimate the number of dominant units from residual variances from regressions of the individual time series on a prespecified number of common factors. In a second step the variances are thresholded to identify the dominant units. In case the dominant units affect only a subset of the other units, the authors propose a multiple testing approach^34^ on the thresholded variances. A disadvantage of the approach is that it requires precise knowledge of the number of common factors and a threshold. Pesaran and Yang^31^ propose an extremum estimator for degree of dominance based on a network or spatial approach. The estimator is based on the ratio between the maximum and of the sum of individual connectivity measures such as a spatial weight matrix.

Brownlees and Mesters^29^ define dominant units with respect to the column norm of the concentration matrix. This implies that in a first step the inverse of the covariance matrix is calculated, limiting the approach to data with more observations than variables. The number of dominant units is then estimated using a growth criterion using a similar approach as the eigenvalue ratio criterion proposed in Ahn and Horenstein^35^. Finally, Gumundsson and Brownlees^33^ identify dominant groups in a VAR using eigenvalues of the autoregressive coefficients. Both methods use the intuitive way to identify dominant units using covariance matrices. However, this is impossible if the number of variables is larger than the number of observations. A partial correlation matrix would be more precise, but hinges on estimation problems and suffers from potential noise in the correlations. A solution is to estimate the covariance matrix using dimensions reduction methods such as the lasso estimator. This approach is followed by Ditzen and Ravazzolo^32^. The authors suggest identifying dominant units using a two-step approach. In the first step a graphical network is estimated using a lasso estimator, following on the lines of Meinshausen and Bühlmann^36^ and Sulaimanov and Koeppl^37^. In a second step the estimated inverse of the covariance matrix is then used to determine the number of factors as in Brownlees and Mesters^29^. A disadvantage of the approach is that it assumes at least one dominant unit. Important to note is that, with exception of Gumundsson and Brownlees^33^, the here mentioned literature originates from factor models. Dominant units are modelled as a special form of a common factor. This is very different from using lasso methods to select the appropriate number of lags in a VAR.

### Rigorous Lasso

This paper follows the approach in Ditzen and Ravazzolo^32^. The authors assume that the data generating process for dominant units is:

$${x}_{it}={u}_{it}, i\in {\Gamma (N}_{d}), \quad t= 1,\dots ,T; \; i= 1, \dots , N$$

and for non-dominant units:

$${x}_{it}=\sum_{j\in\Gamma \left({N}_{d}\right)}{\beta }_{ij}{x}_{jt} +{u}_{it}, \quad i\in {\Gamma (N}_{nd}),\; t= 1,\dots ,T; \; i= 1, \dots , N$$

where ${\Gamma (N}_{d})$ is the set of dominant units, ${\Gamma (N}_{nd})$ the set of non-dominant units and ${u}_{it}$ is a random noise component, allowed to be autocorrelated and heteroscedastic but stationary. The index *i* identifies the cross-sectional unit and *t* the time periods. The identification of dominant units therefore depends on the estimation of ${\Gamma (N}_{nd})$ and the coefficients ${\beta }_{ij}$, which measures the extend unit *j* influences unit *i*. Implicitly the problem requires the estimation of $N(N-1)$ coefficients, whereas the number of observations is NT which is potentially smaller than $N(N-1)$. To solve the high dimensional problem, Ditzen and Ravazzolo^32^ suggest the following optimisation problem which is repeated for each of the *N* cross-sections:

$$\underset{{{\varvec{\beta}}}_{i}}{\mathrm{min}}\frac{1}{T}\sum_{t=1}^{T}{{(x}_{i,t}-{x}_{-i,t}{{\varvec{\beta}}}_{i}^{{\prime}})}^{2}+ \frac{\lambda }{T}\sum_{j=1,j\ne i}^{N}{\psi }_{ij}\left|{\beta }_{ij}\right|,$$

where ${x}_{i,t}$ is a T × 1 matrix containing the observations over time for the *i*-th unit. ${x}_{-i,t}$ is a T x (N-1) matrix containing all other cross-sections. ${{\varvec{\beta}}}_{i}=[{\beta }_{i1},{\beta }_{i2},\dots {\beta }_{ii-1 ,},{\beta }_{ii+1},\dots ,{\beta }_{iN}]$ is a 1 × (N − 1) sparse vector containing coefficients, where the coefficient ${\beta }_{ij}$ measures the effect of unit *j* on unit *i*. In a graphical model, ${\beta }_{ij}\ne 0$ implies that unit *i* and *j* are connected and thus they have a common edge. $\frac{\lambda }{T}\sum_{j}^{N}{\psi }_{ij}\left|{\beta }_{ij}\right|$ is the penalty term with the tuning parameter $\lambda$ and the loading ${\psi }_{ij}$. Both need to be specified prior to estimation, depending on the estimation method. Ditzen and Ravazzolo (2022) find that the rigorous (or plugin) lasso^38–40^ or the adaptive lasso^41,42^ works best to uncover the graphical representation in a framework with dominant units. The rigorous lasso has the advantage that it is data driven and therefore does not require any parametrisation or specification of hyperparameters. In detail, it sets $\lambda =2c \sqrt{N}{\phi }^{-1}\left(1-\frac{\gamma }{2N}\right)$ with *c* a slack parameter, *γ* the probability of the regularisation event and ${\phi }^{-1}$ is the inverse of the cumulative distribution function of the normal distribution^39^. Both parameters are commonly set to *c* = 1.1, *γ* = 0.1/log(N). The penalty loading ${\psi }_{ij}$ is estimated as ${\widehat{\psi }}_{ij}=\sqrt{\frac{1}{T} \sum_{t=1}^{T}{\left({ \dddot{x}}_{itj}{\widehat{\epsilon}}_{it}\right)}^{2} }$ where ${\dddot{x}}_{itj}$ are the deviations from the unit specific means and ${\widehat{\epsilon}}_{it}$ is obtained from an auxiliary regression. Ahrens et al.^40^ show that ${\psi }_{ij}$ can be consistently estimated using autocorrelation and autocorrelation heteroskedasticity robust estimators in the presence of such.

If the adaptive lasso is used, the penalty loadings ${\psi }_{ij}$ are estimated using an unbiased and consistent estimator. Depending on the ratio of variables to observations uni- or multivariate OLS is proven to lead to the desired oracle properties^41,43^. The tuning parameter is commonly specified by either cross-validation or information criteria such as the AIC or BIC. As the rigorous and adaptive lasso both perform similar with respect the identification of the dominant units^32^, we choose the data driven rigorous lasso for our analysis.

The estimated ${\widehat{{\varvec{\beta}}}}_{i}$ are then stacked together ${\widehat{{\varvec{\beta}}}=(\widehat{{\varvec{\beta}}}}_{1},{\widehat{{\varvec{\beta}}}}_{2},\dots ,{\widehat{{\varvec{\beta}}}}_{{\varvec{N}}})^{\prime}$ into a N x N matrix, where the diagonal elements are zero:

$$\widehat{{\varvec{\beta}}}=\left(\widehat{{{\varvec{\beta}}}_{1}},\dots ,\widehat{{{\varvec{\beta}}}_{N}}\right)=\left(\begin{array}{ccccc}0& {\widehat{\beta }}_{\mathrm{1,1}}& \cdots & \cdots & {\widehat{\beta }}_{1,\mathrm{N}}\\ {\widehat{\beta }}_{\mathrm{2,1}}& 0& {\widehat{\beta }}_{\mathrm{2,3}}& \cdots & {\widehat{\beta }}_{2,\mathrm{N}}\\ \vdots & \vdots & \vdots & \vdots & \vdots \\ \vdots & \vdots & \vdots & \vdots & \vdots \\ {\widehat{\beta }}_{\mathrm{N},1}& \cdots & \cdots & {\widehat{\beta }}_{\mathrm{N},N-1}& 0\end{array}\right)$$

Based on Sulaimanov and Koeppl^37^ we obtain the concentration matrix by multiplying the estimated coefficients with the inverse of the unit specific residual variances:

$$\widehat{{\varvec{\kappa}}}=\widehat{{\varvec{D}}}\left(I-\widehat{{\varvec{\beta}}}\right)$$

$$\widehat{{\varvec{D}}}=diag\left({\widehat{\sigma }}_{1}^{-2},\dots ,{\widehat{\sigma }}_{N}^{-2}\right)$$

A feature of this step is that the sparsity of the estimated coefficients carries over to the concentration matrix. It implies that two connected (or dependent) units will be connected in the concentration matrix, while two independent units will be represented by a zero.

Following Brownlees and Mesters^29^ and Ditzen and Ravazzolo^32^ the column norm is used to identify dominant units. Define ${\widetilde{{\varvec{\kappa}}}}_{i}$ as the *i*-th column of $\widehat{{\varvec{\kappa}}}$, then unit *i* is more dominant than unit *j* if the column norm of *i* is larger than of *j*, hence ${||\widetilde{{\varvec{\kappa}}}}_{i}||>{||\widetilde{{\varvec{\kappa}}}}_{j}||$. This implies that a shock to unit *i* has a larger effect to the entire network than a shock to unit *j*. Brownlees and Mesters^29^ and Ditzen and Ravazzolo^32^ suggest identifying the number of dominant units by ordering the column norms by their size and then calculate the growth rate. The maximum of the growth rate defines the estimated number of dominant units $\widehat{k}$:

$$\widehat{k}= \underset{i=1 ,\dots ,\mathit{ N}}{\mathrm{max}}{||\widetilde{{\varvec{\kappa}}}}_{i}||/{||\widetilde{{\varvec{\kappa}}}}_{i+1}||$$

The set of dominant units ${\Gamma (N}_{nd})$ is then defined as the units with the $\widehat{k}$ largest column norms ${||\widetilde{{\varvec{\kappa}}}}_{i}||.$

### Random forests

In order to identify the community-level vulnerability factors of regional drivers, we employ the random forest method as a robust and efficient approach for handling high-dimensional data and uncovering important predictors. High-dimensional data has become a significant focus in recent decades, as technological advancements enable the processing of situations where the number of predictors is larger than the number of data points (i.e., n < p). In many empirical applications, researchers often handpick explanatory variables without relying on a data-driven approach. However, it is not always clear which features are essential and which ones can be dropped without compromising predictive power.

Machine learning, particularly tree-based methods, has proven helpful in identifying relevant predictors in high-dimensional settings with complex interactions. The random forests algorithm^44^ generates multiple trees using randomly selected subsets of observations and features. Averaging these trees results in a smoother and more accurate prediction than a single tree. Random forests are effective in handling sparse settings with many unrelated features^45^, maintaining strong performance even when faced with a large number of features^46,47^.

Random forests offer an efficient approximation of the test error calculated from out-of-bag (OOB) sets through the bootstrap resampling process for each tree. This eliminates the need for cross-validation or a separate test set to obtain an unbiased estimate of the prediction error. As each tree is constructed using a different bootstrap sample from the original data, about one-third of the cases (observations) are left out of the bootstrap sample and not used in the construction of the kth tree. This allows for a test set classification to be obtained for each case in approximately one-third of the trees. The proportion of times that the selected class for the observation differs from the true class over all observations in the OOB set is called the OOB error estimate, which has proven to be unbiased in many tests^48^.

Variable importance measures for random forests have been instrumental in variable selection for various classification tasks in bioinformatics and genetic epidemiology^49,50^. Díaz-Uriarte and Alvarez de Andrés^51^ offer a comparison of random forests and other classification methods for gene expression data analysis. They propose a new gene selection method based on random forests for sample classification using microarray data.

There are several options for evaluating the importance of a specific variable in predictions. One such method is the permutation-based variable importance, which assesses the effect of a variable by randomly reshuffling its data. This method involves taking the original data for the variable, permutating (mixing) the values, and generating "new" data. It then measures the decrease in the model's predictive accuracy after the permutation. If a variable is an important predictor in the model, the model's accuracy will decrease significantly after the variable's permutation. This measure of significance is known as Mean Decrease Accuracy (MDA) and relies on the out-of-bag error estimate.

Note that this approach is distinct from the Mean Decrease Impurity (MDI) method, which evaluates the importance of a variable by calculating the average decrease in impurity (such as Gini impurity) for all trees in the model when the variable is used for splitting. The empirical properties of both criteria have been extensively explored and compared in the statistical computing literature. For example, Archer and Kimes^52^ point out that MDA may behave poorly when correlation increases, which is experimentally tested by Auret and Aldrich^53^ and Tolosi and Lengauer^54^. Auret and Aldrich^53^ show three trends in their simulation study: (1) as the association between a variable and the response increases, the proportion of times that that variable is correctly identified increases; (2) as the correlation of within-group variables increases, the proportion of times the true variable is identified decreases; (3) as the correlation of within-group variables increases, the proportion of times the true group is identified increases.

Strobl et al.^55^ and Hothorn et al.^56^ have suggested a conditional permutation framework to reduce this effect and applied a conditional permutation to their new random forest algorithm forests (conditional forest—CF). Although CF appears to work better for identification of significant variables, it has its own shortcomings specially when the number of trees is large^53,57^. Hence, it is suggested that, when it is feasible, the best practice is to include expert knowledge of the process under consideration in data preprocessing by reducing the presence of correlated variables.

## Results

In this section, we first present the outcomes of the dominance analysis, as outlined in the "Methods" section, to illustrate the spatial network of the influenza-like viral spread where the regions in their dominance are identified and ranked. Following this, we explore the findings of the random forest analysis, which highlights the community-level vulnerability factors of regional drivers.

**Figure 3:**
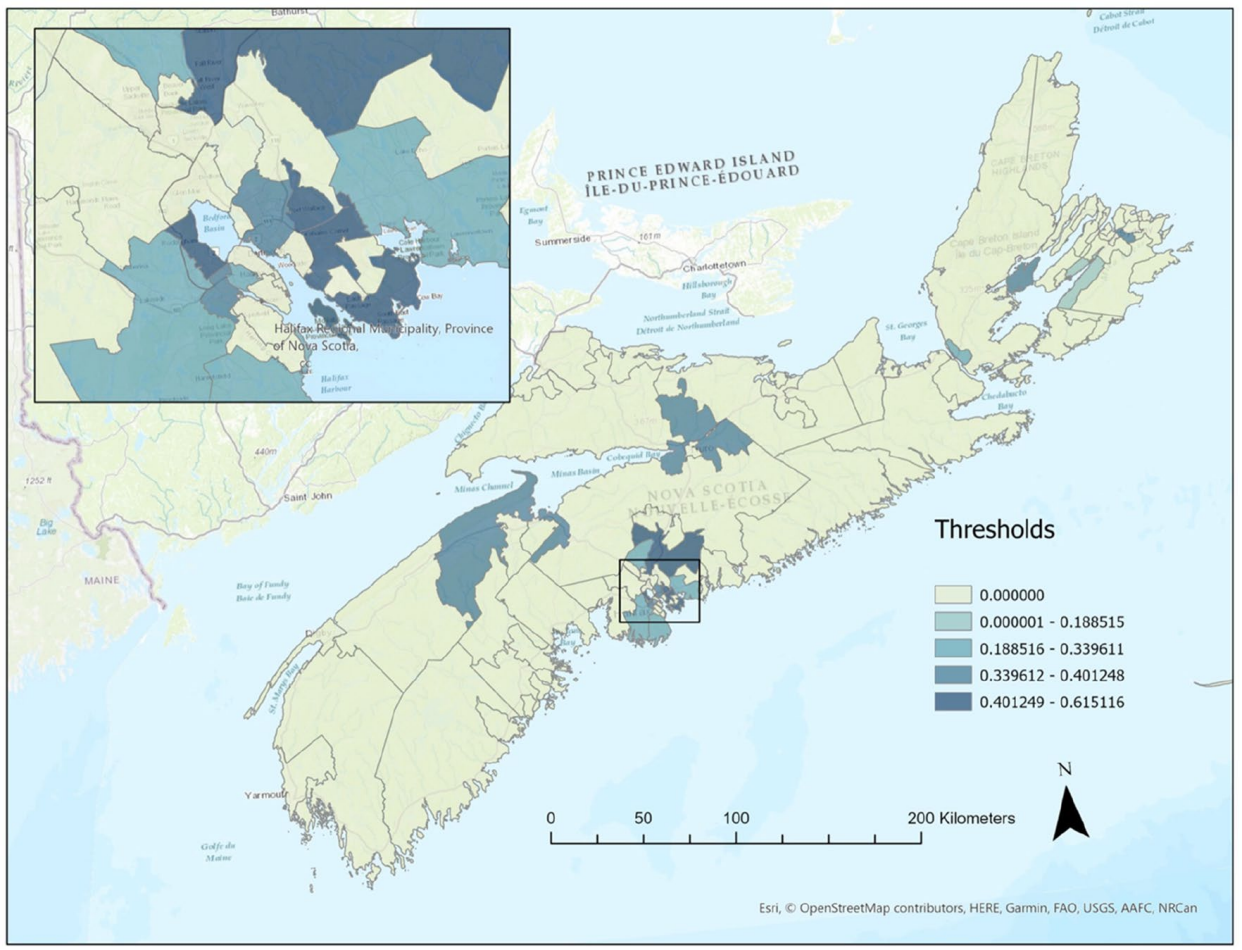
Regional drivers of influenza-like illness in Nova Scotia, Canada. *Note*: the map is created by authors using Esri (Canada) and the map data copyrighted OpenStreetMap contributors and available from https://www.openstreetmap.org.

In our dominance analysis, we utilize the incidence density (or incidence rate), calculated as the number of symptomatic individuals per population, spanning from March 1st to July 1st, 2020. By standardizing the data through first-differencing and scaling, we successfully identify 18 dominant regions among the 77 FSAs, which are illustrated in Fig. 3. The heatmap provides a visual representation of the column norms of rigorous lasso for the 77 FSAs in Nova Scotia, Canada. These norms are used to identify the regional drivers of influenza-like illness. The heatmap illustrates five distinct clusters based on the column norms, with the following thresholds: 58 regions with column norms of 0, 1 region with a column norm of 0.1885, 7 regions with column norms between 0.2742 and 0.3282, 7 regions between 0.3597 and 0.4012 and,5 regions with column norms between 0.4833 and 0.6151.

The varying shades of color in the heatmap reflect the magnitude of the column norms within each FSA with darker shades representing regional drivers. The FSA with a column norm of 0.1885 is not considered a driver region, as it does not meet the criteria for being a dominant contributor to the spread of the virus.

After removing four FSA’s with missing observations, we have 77 FSA’s and 1378 regional predictors. We obtain the results reported in Table 1 based on 2000 runs of the random forest algorithm. This is because a random forest algorithm gets its final estimations as the average of trees using bootstrapped subsamples of observations and features. Therefore, two random forest model estimated from the same data may have slightly different results due the fact that its trees would be different in each model. This particularly true when we have the data that has a very low n/p ratio, which is around 5.0% (77/1378) in our case.

**Table 1:** OOB results of 2000 runs.

Predicted	Actual			
	Dominants	Followers		
Dominants	13	15		
Followers	5	41		
OOB error rate	33.78%			

			95% CI	
PPV		0.46428571	0.45428971	0.47428171
NPV		0.89130435	0.88130835	0.90247635
Specificity—TNR		0.89130435	0.88130835	0.90228035
Sensitivity—TPR		0.72222222	0.71222622	0.73221822
Balanced Accuracy		0.80676329	0.79676729	0.81724929

*PPV* positive predictive value, *NPV* negative predictive value, *TNR* true negative rate, *TPR* True positive rate.

After eliminating a few rural FSAs with a very low population density and one outlier region, we include 74 total FSAs (18 "Dominants", 56 "Followers") in our initial application. Although the class balance is not skewed drastically (18/56 = 0.321), we use a stratified random forest algorithm to reduce the bias in each split. We also use the default setting of randomly selected features ("mtry" = $\sqrt{p}$= 37) in each tree. The OOB results are shown in Table 1. Note that the confusion table is obtained by averaging 2000 runs and the values rounded down to the nearest integer. All the results indicate a decent prediction accuracy of our initial application without preprocessing and explicit training.

In our study, the outcome variable is categorical (Y = 1 for spreader, 0 otherwise). The random forest algorithm calculates the predicted probability of success (Y = 1) and uses a fixed cut-off threshold (c) to determine the prediction outcome. The predictive accuracy of the model depends on the cut-off threshold (c) and can be summarized by the Area Under Curve (AUC) of Receiver Operating Characteristics (ROC), which illustrates the trade-off between True Positive Rate (TPR) and False Positive Rate (FPR).

Using the out-of-bag (OOB) probabilities averaged over 1500 trees, we determine the optimal cut-off threshold (c) and calculate the AUC for each run. After averaging over 2000 runs, the AUC is found to be 74.6%, with a standard error of 0.019051. By setting the number of selected features ($\sqrt{p}$) as a hyperparameter and performing a grid-search with a fivefold cross-validation process repeated 5 times, the OOB AUC improves to 81.56%, with a standard error of 0.03787.

These results reflect the out-of-sample prediction accuracy. When the trained model is applied for in-sample predictions using the entire data, the AUC increases to 89.11%, indicating good internal validity. We also implement preprocessing operations to reduce highly correlated predictors and near-zero-variance features. Although the results do not significantly improve after preprocessing, the selection of important predictors benefits from this process, which we will discuss next.

To evaluate the importance of variables in our predictions, we employ Mean Decrease Impurity (MDI) alongside a two-step preprocessing method. As we discussed in the "Methods" section, permutation-based importance measures, such as Mean Decrease Accuracy (MDA), can be biased due to correlated predictors. To address this issue, we choose MDI, which provides a more robust measure of variable importance by considering the decrease in node impurity across all trees in the random forest.

The two-step preprocessing involves first removing 126 variables with near-zero variance to prevent a few samples from having undue influence on the model. Second, we identify and reduce within-group correlations between predictors to further address potential bias in MDI and MDA. After setting the cut-off correlation coefficient at 0.85, we remove 679 additional variables, resulting in 375 regional predictors in our algorithms. The census file's hierarchical aggregation and gender partitions account for many of the removed predictors. We tested various cut-off points between 0.85 and 0.99 and found that more than 60% of the top predictors remain consistent, with predictive accuracy staying about the same, validating the selection of removed variables.

**Figure 4:**
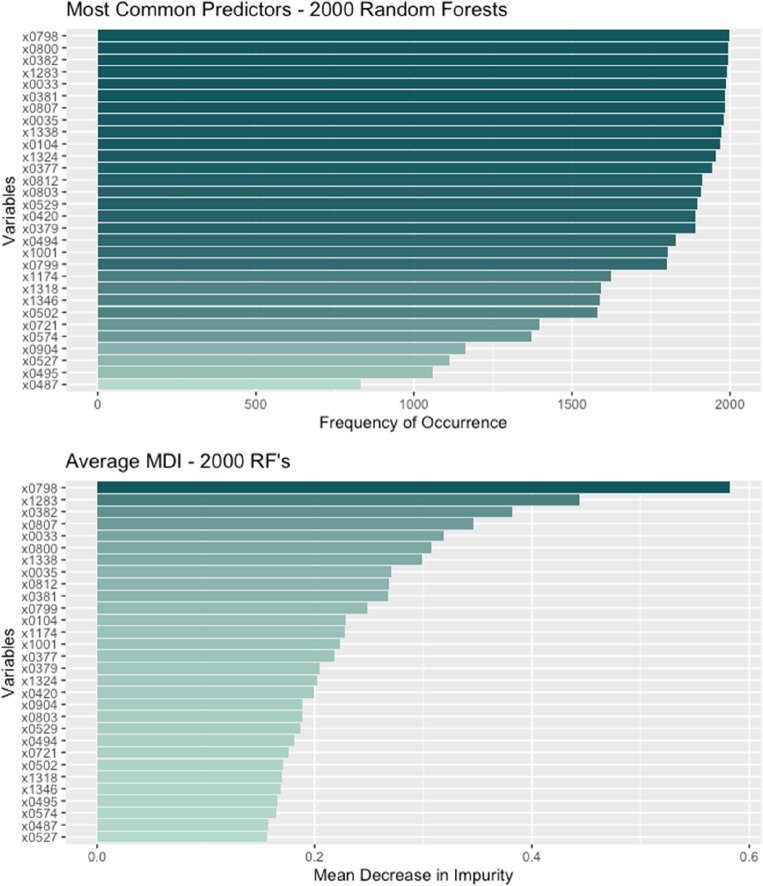
Variable importance measures over 2000 random forests. *Note*: The names of variable codes are given in Table 2.

Figure 4 displays the top 30 predictors, ranked by their selection frequency in 2000 runs (top plot) and by their average MDI across these runs (bottom plot). Both plots feature the same list of predictors, highlighting the consistency of the top variables and the effectiveness of the MDI measure in identifying the most important predictors for our model.

Variable importance methods typically provide global measures, rather than local or directional ones. Directional local variable importance measures based on ceteris paribus profiles can be obtained through partial dependence profiles at each prediction point, such as for each major FSA. Several tools exist for instance-level explorations and understanding local-level profiles^58^. However, in this study, we employ a simple difference-in-means comparison for selected predictors, as shown in Table 2, for two reasons.

**Table 2:** Differences-in-means comparisons for selected predictors.

Code	MDI	Followers	Dominants	Names
×0798	0.582	50.6	61.2	Homeowners with a mortgage (%)
×1283	0.444	40.1	42.7	Weeks worked
×0382	0.382	64.2	70.5	With employment income (%)
×0807	0.346	764.9	901.8	Median monthly rent $
×0033	0.319	64.8	67.8	People between 15 and 65 (%)
×0800	0.308	793.9	1080.4	Med. monthly shelter cost—owners
×1338	0.299	218.2	727.5	Using public transit to work
×0035	0.271	2.4	1.7	People 65 + (%)
×0812	0.268	20.9	36.9	People with Inuit origin
×0381	0.268	81.6	87.1	Total market income $
×0799	0.249	11.6	12.0	Spent 30% + inc. on shelter (%)
×0104	0.228	211.0	146.7	People speak French at home
×1174	0.228	1.7	7.2	People in military service
×1001	0.223	4.6	17.2	People with African origin
×0377	0.218	8111.6	5910.1	Median government transfers $
×0379	0.205	28,918.8	33,548.6	Median employment income $
×1324	0.202	426.0	904.7	People working in public sector
×0420	0.199	66.0	70.8	With employment income (%)
×0904	0.189	2.9	11.4	People with Caribbean origin
×0803	0.189	218,969.0	267,140.6	Average value of dwelling $
×0529	0.187	17.6	12.9	People 65 + with low-income
×0494	0.181	88,184.2	91,594.5	Med. income—families with kids $
×0721	0.176	13.8	26.1	People with aboriginal ancestries
×0502	0.171	26,360.5	29,776.0	Med. income—noneconomic fam. $
×1318	0.170	254.2	444.4	People in waste management ind
×1346	0.168	298.6	424.2	People travel to work 45–59 min
×0495	0.166	3.8	3.9	Average family size—with kids
×0574	0.165	14.4	34.2	Immigrants from Americas (other)
×0487	0.158	2.7	2.8	Average family size—Overall
×0527	0.156	27.1	21.9	People with low-income 0–17

First, our aim is not to uncover causal relationships, which would be better achieved with semi-parametric applications like multiple adaptive regression splines (MARS). Second, extracting local variable importance is an active research area, and many of the tools developed for understanding local and directional effects in random forests rely on strong assumptions, such as the independence between predictors.

Table 2 presents the key predictors associated with regional drivers of the viral spread. A more discussion of these findings is provided in the next section.

## Discussion

In the "Results" section, we delved into the identification of "regional drivers" of viral spread through a dominant unit analysis. It might be assumed that the order of column norms would correlate with the level of infection density in each Forward Sortation Area (FSA), implying that areas with high infection density could serve as primary sources for regional spreading. However, our analysis revealed a low correlation (0.31) between the column norms selected by rigorous lasso and the infection densities in each dominant FSA. This finding suggests that regions with low infection density could still act as influential regional drivers, while areas with high-density hotspots may play a more submissive role in viral spread.

The essential distinction in pinpointing regional drivers is that our dominant unit analysis accounts for the temporal dynamics of viral spread within its network structure, as opposed to infection densities, which only represent cumulative sums and cross-sectional differences.

The existing body of research on characterizing spatiotemporal trends in the spread of viral pathogens is limited. Most of the literature focuses on the impact of influenza-like illnesses in one area on its neighboring regions^59,60^, utilizing personal contact data. To overcome data limitations, Qiu et al.^61^ proposed the concept of a spatiotemporal route to demonstrate potential transmission directions and the magnitudes of those effects. This concept is based on time-lagged associations among influenza surveillance data from different locations, using vector-autoregressive models (VAR). Wang et al.^62^ later employed this method to predict the spread of viral infections.

While VAR models are effective in forecasting complex spatiotemporal networks, they struggle to reveal the underlying structure of these networks, particularly when it comes to identifying dominant units within panel data. Our machine learning-assisted approach shares some similarities with the work of Qiu et al.^61^, but there are key differences that set our method apart. Most notably, our approach aims to identify which units are dominant, whereas Qiu et al.^61^ view all non-zero connections as channels for spreading influenza, without considering their actual importance. In addition, Qiu et al.^61^ base their analysis on the spread of influenza being dependent on time, while our approach adopts a time-independent perspective. These distinctions allow us to gain a deeper understanding of the underlying dynamics of regional drivers in viral spread and develop more effective strategies to tackle outbreaks.

In the "Results" section, we also presented findings related to the characteristics of driver regions. Table 2 sheds light on key factors that distinguish driver regions from others in terms of viral spread, suggesting a multifaceted interplay of social, economic, and demographic factors that contribute to regional transmission dynamics.

Socioeconomic factors such as homeownership, mortgage status, longer working weeks, and higher employment income might signify increased mobility and social interactions among these populations. Individuals in driver regions may have more work-related obligations or social events that could lead to increased contact with others, contributing to the spread of the virus.

Living conditions and urban settings, as indicated by higher median monthly rent and a higher percentage of people aged 15–65, may suggest denser residential areas with a larger economically active population. These conditions could increase the likelihood of close contact with others in shared spaces such as apartments, elevators, or public areas, thus promoting viral transmission.

Transportation factors, such as the use of public transit and longer travel times to work, could expose individuals in driver regions to higher risks due to increased contact with others during their commute. This increased exposure, in turn, might contribute to a higher transmission rate in these regions.

The demographic composition of driver regions, specifically the presence of people with Inuit, African, Caribbean, or Latin American origins, may indicate shared socio-economic conditions, occupational risk factors, or living arrangements that influence transmission patterns. For instance, some communities might have larger households, multi-generational living arrangements, or higher rates of essential worker occupations, which could increase the potential for viral spread.

Public sector employment, military families, and people working in waste management industries might face specific job-related risk factors or living conditions that contribute to regional transmission dynamics. These groups may have unique work environments or living arrangements that increase their exposure to the virus, such as shared barracks, on-base housing, or frontline work conditions.

In summary, the factors identified in Table 2 paint a complex picture of the regional drivers of influenza-like illness. Understanding these interrelated factors is crucial for developing targeted public health interventions that address the unique challenges faced by driver regions in containing the virus. Further research is needed to explore the interactions between these factors and elucidate the underlying mechanisms driving regional transmission dynamics.

## Concluding remarks

Studies exploring outbreaks through the lens of social geography offer valuable tools for understanding the spatial and temporal determinants of disease spread. It is well-established that social, geographic, and economic factors influence the transmission rate of infectious diseases. This study builds upon prior research by examining how contextual factors contribute to the spatial distribution of viral spread in several innovative ways.

The unique dataset generated by the local public response to the COVID-19 pandemic in Nova Scotia resembles controlled clinical trials. We apply a novel method related to recent literature on granular time series to investigate the formation of spatial dependence in the network of regions. By analyzing the temporal dynamics in the data, we identify and rank each region's dominance in the spatial transmission network. This new method, when applied to epidemiological surveillance, reveals “dominant regional drivers” and their associated socio-spatial predictors.

Our study identifies 18 dominant regional drivers among 112 regions and discovers significant space-specific characteristics associated with them. These regional drivers are characterized by their community-level vulnerability, such as demographic and economic factors. We suggest that predictive detection and spatial analysis be incorporated into population-based surveillance strategies to enhance early case detection and optimize the allocation of healthcare resources.

Future research can build upon these findings by expanding the scope to other infectious diseases or evaluating the impact of various public health interventions on spatial distribution. Longitudinal analysis could be employed to assess temporal changes in dominant regional drivers and their associated socio-spatial predictors, helping us understand the evolving nature of transmission networks over time. Additionally, the integration of real-time mobility data, social media data, or other novel sources of information could lead to a more comprehensive understanding of the drivers of disease spread and their interaction with socio-spatial factors. By exploring these areas in future research, we can continue to refine our understanding of the socio-spatial factors influencing the spread of infectious diseases, ultimately contributing to the development of more targeted and effective public health interventions.

## Data Availability

This study uses a confidential and administrative dataset for COVID-19 tests obtained from Nova Scotia Health Authority. Its anonymized version aggregated for each FSA level is available for dominance analysis. The census data can be obtained from Census Analyzer: http://dc1.chass.utoronto.ca/census/index.html.

## Abbreviations

AIC: Akaike information criterion
AUC: Area under curve
BIC: Bayesian information criterion
CF: Conditional forest
FPR: False positive rate
FSA: Forward sorting area
MARS: Multiple adaptive regression splines
MDA: Mean decrease accuracy
MDI: Mean decrease impurity
NPV: Negative predictive value
NSHA: Nova Scotia health authority
OOB: Out-of-bag
PPV: Positive predictive value
RF: Random forest
RNS: Research Nova Scotia
ROC: Receiver operating curve
TNR: True negative rate
TPR: True positive rate
VAR: Vector autoregression
